# Editorial: How can wearable robotic and sensor technology advance neurorehabilitation?

**DOI:** 10.3389/fnbot.2022.1033516

**Published:** 2022-10-11

**Authors:** Shuo-Hsiu Chang, Shih-Chiao Tseng, Hao Su, Gerard E. Francisco

**Affiliations:** ^1^Department of Physical Medicine and Rehabilitation, University of Texas Health Science Center at Houston, Houston, TX, United States; ^2^Neurorecovery Research Center, TIRR Memorial Hermann, Houston, TX, United States; ^3^Department of Physical Therapy, University of Texas Medical Branch, Galveston, TX, United States; ^4^Department of Mechanical and Aerospace Engineering, North Carolina State University, Raleigh, NC, United States

**Keywords:** wearable robotics, wearable sensors, rehabilitation, neurorehabilitation, rehabilitation technology

## Introduction

Current rehabilitation rests on two approaches: restorative and compensatory. The restorative approach aims to re-establish lost functions *via* a specific mechanism called neuroplasticity, defined as the ability of the nervous system to regenerate and reorganize its structure, functions, and connections. Neuroplasticity is activity-dependent, where repetitive, targeted, consistently challenging exercise facilitates specific functional recovery *via* restoring impaired neural circuits, growing new neural circuits, and generalization to real-world settings (Kelly et al., 2006; Dietz and Fouad, 2014; Hylin et al., 2017). On the other hand, the compensatory approach is based on the principle of adaptability, which does not focus on changing and restoring the physical structure or body function. Instead, it is designed to minimize the effect of deficits and recover a degree of function by developing internal and external strategies in which the individuals use residual, intact ability to adapt to the changes in their body functions post injuries or diseases. Assistive rehabilitation technology offers an opportunity to enhance the user's experience by repeatedly practicing specific tasks that are precisely controlled *via* real-time kinetic or kinematic feedback and assistance as needed to minimize functional deficits and environmental barriers (Grimm et al., 2016). Hence, the use of rehabilitation technology can change the individual's experience impacting the ability of functional recovery *via* the mechanism of neuroplasticity and minimizing the deficits *via* adapting changes to their body structures and functions post-disease or injury (Grimm et al., 2016).

Modern technological advances in wearable technologies in health care have made it possible to augment patient clinical outcomes (Afzal et al., 2020; Terranova et al.; Edwards et al., 2022; Werner et al.), create an effective continuum of rehabilitation (Ahmed et al., 2022; Garnier-Villarreal et al., 2022), and promote independence (Bützer et al., 2021; Liu et al., 2022) for persons after neurological injuries. In particular, the technology allows more intensive and tailored patient rehabilitation activities and services (increasing the amount and quality of therapy that can be administered and supervised) and enable all the involved actors in the team (e.g., physicians, therapists, bioengineers and others) to design patient-centered and custom intervention collaboratively. Moreover, technology can transform rehabilitation from a one-on-one human resource intensive treatment that can only be provided in specialized centers to a technology-driven, remotely-supervised and widely accessible enterprise (Moulaei et al., 2022). Given the increased costs associated with long-term rehabilitation and the difficulty in providing appropriate duration and intensity of rehabilitation services required to manage disability, cost-effective development of robotic rehabilitation is greatly warranted.

In this special issue, we have collected ten articles on the following themes.

### Neuromuscular adaptation mechanisms using wearable robotics

Le et al. investigated the cortical activity associated with executive resource allocation during a motor task with an assistive upper limb exoskeleton robot.

### Design and experimentation of novel robotics

Zhang and Collins explored the possibility of optimizing the post-stabilization real-time control performance on lower-limb legged robots driven by series elastic actuators. Chen et al. provided a novel proof-in-conception prosthesis myocontrol approach focused on hip disarticulation amputees and investigated the performance of the gait phase decoder fusing bilateral neuromechanical signals. Porciuncula et al. investigated the feasibility and rehabilitative potential of applying propulsion-augmenting exosuits as part of an individualized and progressive training program to retrain faster walking and the underlying propulsive strategy. Wang et al. investigated different assistance patterns' effectiveness in improving the walking economy on a lightweight cable-driven ankle exoskeleton.

### Evidence of clinical trials using robotics

Brinkemper et al. investigated whether an improvement in physiological gait can be demonstrated in addition to the functional parameters after treatment with a robot suit in persons with acute and chronic spinal cord injury (SCI). Zieriacks et al. investigated the impact of exoskeleton-assisted body weight-supported treadmill training on functional and motor recovery in post-acute neurorehabilitation in persons with acute and chronic SCI. Treviño et al. investigated which factors predict the improvement in Functional Independence Measure score after robotic exoskeleton rehabilitation in persons with cerebrovascular accidents or traumatic brain injuries. Finally, Casas et al. investigated the effectiveness of 8-week home-based therapy using an upper limb robot in persons with chronic stroke.

### Clinical and administrative experience in the clinical implementation of wearable robotics in healthcare settings

Nolan et al. presented a multicenter investigation on the utilization of a robotic exoskeleton for overground walking in persons with acute and chronic stroke.

## Conclusion

These articles added much knowledge in supporting the feasibility and safety of wearable and robotic technology, i.e., wearable upper and lower limbs exoskeletons to facilitate neuromotor and functional recovery in persons with sensor-motor impairments and disabilities. Moreover, this special issue illustrates not only the broad efforts of adapting technology into rehabilitation care but also the future research direction of the field. The articles reflect the current emphasis on the hardware and software design and early phases of clinical trials in using the technology. While the field needs more multidisciplinary conceptual and collaborative efforts (i.e., the team with clinicians and engineers), we hope the knowledge will further be developed into areas including patient-centered, value-based, and accessible rehabilitation care, considered an exciting future trajectory for neurorehabilitation.

## Author contributions

S-HC and S-CT wrote the first draft of the editorial. HS and GEF contributed to manuscript revision, read, and approved the final manuscript. All authors contributed to the conception of the special Research Topic and took individual responsibility for editing separate articles.

## Funding

This work was supported by Memorial Hermann Foundation to GEF and NIDILRR under Grant 90DPGE0011 to HS and S-HC.

## Conflict of interest

The authors declare that the research was conducted in the absence of any commercial or financial relationships that could be construed as a potential conflict of interest.

## Publisher's note

All claims expressed in this article are solely those of the authors and do not necessarily represent those of their affiliated organizations, or those of the publisher, the editors and the reviewers. Any product that may be evaluated in this article, or claim that may be made by its manufacturer, is not guaranteed or endorsed by the publisher.
